# Using workshops to develop theories of change in five low and middle income countries: lessons from the programme for improving mental health care (PRIME)

**DOI:** 10.1186/1752-4458-8-15

**Published:** 2014-04-30

**Authors:** Erica Breuer, Mary J De Silva, Abebaw Fekadu, Nagendra Prasad Luitel, Vaibhav Murhar, Juliet Nakku, Inge Petersen, Crick Lund

**Affiliations:** 1 Alan J Flisher Centre for Public Mental Health, Department of Psychiatry and Mental Health, University of Cape Town, 46 Sawkins Road, Rondebosch 7700, Cape Town, South Africa; 2 Centre for Global Mental Health, London School of Hygiene and Tropical Medicine, Keppel Street, London WC1E 7HT, UK; 3 Department of Psychiatry, Addis Ababa University, College of Health Sciences, School of Medicine, PO Box 9086 Addis Ababa, Ethiopia; 4 King's College London, Institute of Psychiatry, Department of Psychological Medicine, Centre for Affective Disorders and Affective Disorders Research Group, London, UK; 5 Transcultural Psychosocial Organization Nepal, Baluwatar, Box 8974, Kathmandu, GPO, Nepal; 6 Sangath, HN – 6, Rishi Nagar, Char Imli, Bhopal, Madhya Pradesh, India; 7 Butabika National Mental Hospital, Kampala, Uganda; 8 School of Psychology, University of KwaZulu-Natal, Howard College Campus, Durban 4000, South Africa

**Keywords:** Theory of change, Programme evaluation, Programme design, Health planning, Mental health

## Abstract

**Background:**

The Theory of Change (ToC) approach has been used to develop and evaluate complex health initiatives in a participatory way in high income countries. Little is known about its use to develop mental health care plans in low and middle income countries where mental health services remain inadequate.

**Aims:**

ToC workshops were held as part of formative phase of the Programme for Improving Mental Health Care (PRIME) in order 1) to develop a structured logical and evidence-based ToC map as a basis for a mental health care plan in each district; (2) to contextualise the plans; and (3) to obtain stakeholder buy-in in Ethiopia, India, Nepal, South Africa and Uganda. This study describes the structure and facilitator’s experiences of ToC workshops.

**Methods:**

The facilitators of the ToC workshops were interviewed and the interviews were recorded, transcribed and analysed together with process documentation from the workshops using a framework analysis approach.

**Results:**

Thirteen workshops were held in the five PRIME countries at different levels of the health system. The ToC workshops achieved their stated goals with the contributions of different stakeholders. District health planners, mental health specialists, and researchers contributed the most to the development of the ToC while service providers provided detailed contextual information. Buy-in was achieved from all stakeholders but valued more from those in control of resources.

**Conclusions:**

ToC workshops are a useful approach for developing ToCs as a basis for mental health care plans because they facilitate logical, evidence based and contextualised plans, while promoting stakeholder buy in. Because of the existing hierarchies within some health systems, strategies such as limiting the types of participants and stratifying the workshops can be used to ensure productive workshops.

## Background

Mental health services remain inadequate in low and middle income countries (LMIC). They are marked by low financial investment, insufficient human resources and lack of political priority and planning for mental health care [1,2]. In order to expand and improve access, it is imperative that mental health is integrated into primary health care and other health platforms as well as into the services provided by other sectors including education, social services, justice and labour [3]. Although evidence exists for individual evidence based interventions, less is known about how they can be integrated into existing health services [4]. Engaging key stakeholders in participatory planning for mental health services is critical to develop such services and resources, get local and national stakeholder buy-in, and develop plans that are contextually appropriate [5,6].

Theory of Change (ToC) is a participatory theory driven approach to programme design and evaluation whose underlying principle is to improve our understanding of how and why a programme works [7]. This is achieved through the development of a ToC, or programme theory, which describes the causal pathways through which a programme is hypothesised to have an effect. The ToC is often developed in consultation with key stakeholders in ToC workshops or interviews, document review or programme observation [8]. Social science, management, sociological or other formal theories are inserted into the framework to explain why and how the causal pathways operate [8,9]. The ToC is often displayed visually as a ToC map [10].

The ToC approach was developed from theory driven evaluation approaches which include the logical frameworks and logic models [11] and has been influenced by informed social action approaches [12]. Although often used exclusively as an evaluation tool, Connell and Kubisch in one of the seminal articles on ToC proposed that it be used both in for programme development and evaluation [13].

Interest in the ToC approach has grown recently in the development and NGO sector used by agencies such as DFID, Oxfam, and Comic Relief [14] for both program design and evaluation. Despite the abundance of guidelines on how to develop a ToC [10,15,16] and its widespread use there are few published case reports of their application in the academic literature and the majority of these describe the use in evaluation of programmes and not their design. There are only a few examples in the academic literature describing the role of ToC in the planning of complex health interventions [17,18]. These include the use of ToC in the development and evaluation of mental health systems of care for children and adolescents in the US [19,20]. Results from these experiences show that ToC can be used effectively as a planning tool for implementation as well as provide a framework for evaluation [20]. In addition, using ToC provides a mechanism for consensus building amongst stakeholders and a shared service delivery strategy.

There is very little detail published on how ToCs have been developed. Methods of ToC development reported in the literature include review of programme documentation [21], interviews and focus group discussions with key stakeholders [17,22,8], using existing theory or research [23,24] and ToC workshops [8] but few describe their methods in enough detail to replicate the ToC development.

Proponents of ToC advocate for the use of ToC workshops to develop ToCs as they allow participation of various stakeholders who can share knowledge, debate specific aspects of the ToC, articulate assumptions, and assess the feasibility of the intended interventions in the specific context [8,13]. For example, Mason and Barnes (2007) used ToC workshops with key stakeholders to develop a ToC for the evaluation of the New Children’s Fund, a multi-agency collaboration to deliver preventive services for children. During the workshops, they explored the needs of the target group, the short, medium and long terms outcomes the programme was working to address, the activities through which the outcomes could be achieved, the rationale of the activities, and the local and national policy context. However, few additional examples of ToC workshops have been published in the academic literature [25] and, to our knowledge, none use ToC workshops to develop an intervention within a health system in LMIC.

**Figure 1:**
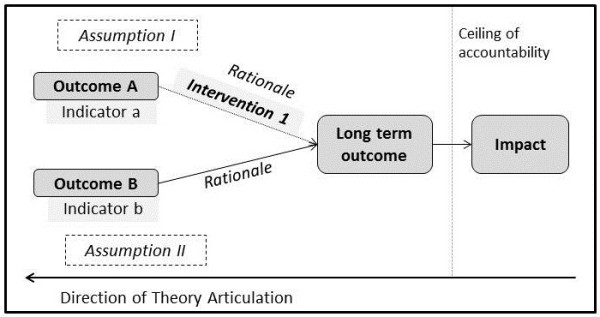
Elements of a Theory of Changes (adapted from Andersen 2004).

The majority of the guidance on how to conduct ToC workshops has been developed by funding and development organisations which outline how ToC workshops can be conducted. A common approach starts with stakeholders reaching agreement on the intended *impact*, then working backward to determine the intermediate and short term *outcomes* necessary and sufficient to achieve the intended impact [10,26]. These outcomes are operationalized by identifying *indicators* for each outcome which will determine whether the outcome has been achieved. In addition, the evidence base or *rationale* of how one outcome leads to the next is articulated and whether an *intervention* is required to achieve this. Stakeholders are encouraged to articulate the *assumptions* underlying the theory as well as to decide a *ceiling of accountability* where the programme is no longer directly responsible for the outcomes achieved. The ultimate ToC should be plausible, do-able and testable [13] and can be represented graphically in a ToC map (Figure 1).

### The programme for improving mental health care (PRIME)

The Programme for Improving Mental Health Care (PRIME) is a multi-country research programme which aims to provide evidence for how to integrate mental health into primary care by developing, implementing and evaluating district level mental health care plans (MHCPs) for priority disorders [27]. It is working in pilot districts or sub-districts in five LMIC, namely in Sodo, Ethiopia; Sehore, India; Chitwan, Nepal; Dr Kenneth Kuanda, South Africa; and Kamuli, Uganda (Table 1). Mental health service resources vary considerably across the district sites. Still, all countries face health systems and contextual challenges [28]. Within each district, specific packages of mental health care made up of several interacting components have been developed for implementation within three levels of the health system: healthcare organisation, health facility and community. The PRIME MHCPs target three priority disorders: depression, alcohol use disorders and psychosis, with the addition of epilepsy in Ethiopia, Nepal and Uganda. One of the key principles of PRIME is a partnership between researchers and the Ministries of Health in each of the PRIME countries. As part of this partnership, the human resources for the implementation of PRIME are largely provided by the Ministries of Health while the researchers provide training, technical support and evaluation [29]. As such, the PRIME MHCPs meet the criteria for complex interventions as outlined by Craig et al. [29] including multiple groups of stakeholders and organisational levels targeted by the intervention. The intervention achieves multiple outcomes through several causal strands.

**Table 1:** **PRIME district sites adapted from Lund et al. **[29]

**Country**	**World bank region**^**1**^	**World bank income classification**^**1**^	**Gross national income per capita (USD)**^**1**^	**PRIME District/sub-district**	**Population**^**2**^	**Socio-economic characteristics**^**2**^	**Number of Health Facilities**^**2**^	**Number of MH specialists**^**2**^
**Ethiopia**	Sub-Saharan Africa	Low Income	400	**Sodo**	165,000	Literacy rate = 22%; 90% rural	0 hospitals, 1 district health bureau, 7 community health centers, 52 health posts	None
**India**	South Asia	Lower middle income	1410	**Sehore (Madhya Pradesh state)**	1,311,008	Literacy rate: 71% 81% rural	2 hospitals, 8 community health centers, 15 primary health clinics	1 part-time psychiatrist, 1 psychologist
**Nepal**	South Asia	Low income	540	**Chitwan**	575,058	Literacy rate = 70% 73% rural	152 sub –health centers	2 Psychiatrists
**South Africa**	Sub-Saharan Africa	Upper Middle Income	6960	**Kenneth Kaunda (North West Province)**	632,790	Literacy rate: 88% 14% rural	2 hospitals, 4 primary health centers, 5 health posts	1 Psychiatrist,
								1 Psychologist
**Uganda**	Sub-Saharan Africa	Low Income	500	**Kamuli**	740,700	Literacy rate: 62% 3% rural	41 sub-health posts	1 Psychiatric Clinical Officer

^1^Countries and Economies [http://data.worldbank.org/country/] [30].

The PRIME MHCPs have been developed for each district through formative work including reviews of the literature, a situational analysis of mental health care in the district [28], semi structured interviews and focus group discussions with stakeholders [31]. As part of the development of the PRIME MHCPs we used a ToC approach which involved the development of a PRIME cross-country and district specific ToCs.

This paper describes how the district specific workshops were used in the planning stages of PRIME. Specifically, we describe the overall structure and stakeholder composition of the workshops and the facilitators’ experiences of how stakeholders contributed to the three purposes of the ToC workshops. The purposes were to 1) develop a logical evidenced based ToC map, 2) inform the development of a contextualised mental health care plan; and, 3) obtain the buy-in of key stakeholders. We further describe how these purposes were achieved within a hierarchical health system and potential approaches to and limitations of mitigating the effects of this hierarchy.

## Methods

### The ToC process in PRIME

The ToC process began by developing an initial PRIME cross-country ToC in a workshop attended by 15 key PRIME partners, including two representatives from each PRIME country team, in Goa, India in October 2011. The workshop aimed to both introduce the PRIME partners to the ToC approach and to develop a PRIME cross country ToC as a framework for the district level MHCPs.

**Figure 2:**
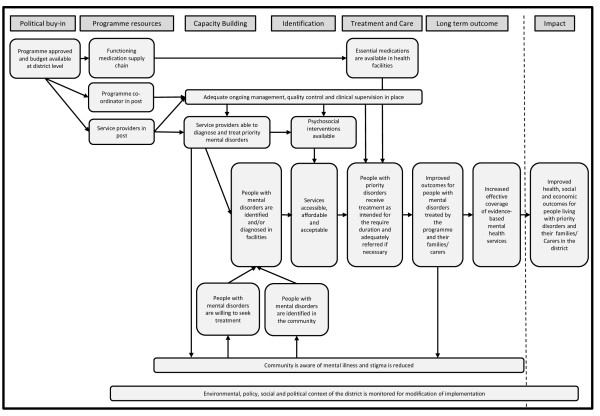
An abridged version of the outcomes and impact of the PRIME Cross Country Theory of Change.

The ToC developed during this workshop identified the intended impact of the PRIME intervention, namely improved health, social and economic outcomes in people living with the priority mental disorders in the selected districts of PRIME. The workshop participants identified the anticipated short, medium and long term goals required to achieve the impact across the three levels of the health system. The outcomes were identified in the following domains: political buy-in, programme resources, capacity building, identification and diagnosis of mental disorders and service delivery. Participants also identified assumptions and gaps in knowledge which informed the development of the formative research questions to develop contextualised MHCPs in each district. The ToC was then refined and modified by members of the PRIME consortium over the following year. An abridged version of the ToC showing the outcomes only is illustrated in Figure 2. The overarching cross country process of ToC development in PRIME, the resulting ToC map and the influence of the ToC on the PRIME cross country evaluation design will be described in detail in a subsequent paper.

Following this, each PRIME country team conducted at least two ToC workshops to assist with the development of the district specific ToCs. These workshops aimed to: 1) develop a logical evidenced based ToC, 2) inform the development of a contextualised mental health care plan; and, 3) to garner the buy-in of key stakeholders. The resulting ToCs were used as a ‘blueprint’ for the PRIME MHCPs which were developed further using results of the PRIME situational analysis, formative work, costing tool and literature reviews. The ToCs were used to validate and expand on the PRIME cross country ToC which was used as a framework to evaluate the effectiveness of the plan following implementation. The structure, number, composition and process of the workshops was determined by PRIME country teams in line with brief cross country guidelines in conjunction with Andersen’s guidelines [10].

Stakeholders were defined as those involved in the implementation of the program, served or affected by the program or using the evaluation results [32]. They were purposively sampled and recruited at the discretion of country teams who aimed to balance a productive workshop with the hierarchical nature of their respective health systems. Participants included diverse stakeholders such as district health service managers, primary health care service providers, district mental health coordinators, members of the community leaders, mental health specialists, national level policy makers and mental health service users.

### Data collection and tools

Data about the ToC workshops were collected from a number of sources. First we collected process documentation from all workshops produced by the PRIME country teams in English. This included minutes or workshop reports which reported on the key content and structure of the workshops and participant lists.

Secondly, we conducted 5 individual and 5 joint semi-structured interviews with 9 facilitators of the ToC workshops (4 principal investigators and 5 project co-ordinators) following both the preliminary and final workshops. The decision to conduct joint interviews with 2–3 facilitators from one country, or individual interviews, was made by country principal investigators. The interviews were designed to elicit the facilitators’ experiences of the workshops and stimulate discussion on the practical aspects of how the workshops were conducted namely stakeholder composition, workshop structure, group dynamics, the usefulness of the process and to generate lessons for future use in intervention development.

All interviews were telephonically or face-to-face conducted by the first author (EB) in English and transcribed by a professional transcriber familiar with health research. The transcripts were checked for accuracy by EB. Additional information was gained through email correspondence, informal discussions and presentations at PRIME consortium meetings as well as direct experience of the workshops by co-authors.

### Data analysis

A framework analysis approach was used to analyse the process documentation and interview transcripts [33]. This method was developed for applied policy research and contains five key stages: familiarisation, identifying a thematic framework, indexing, charting, mapping, and interpretation. Qualitative data software, NVivo9, was used to assist with the analysis [34].

Following familiarisation with the process documentation and interview transcripts, a coding framework was developed by EB based on the semi structured interview guide. The main themes included workshop structure, participants, dynamics and emerging themes. A framework matrix was generated using the themes: workshop structure; participants and dynamics; and their 25 subthemes which mapped onto the X axis of a spreadsheet. The 13 ToC workshops were mapped onto the Y axis. The coded data in each cell were summarised to reflect the content. The data within each column or sub-theme was compared across ToC workshops and interpreted. As the data were compared additional salient themes emerged. Following the main analysis by EB, the results were summarised and validated by co-authors who were part of the country workshops.

The study was approved by the Human Ethics Research Committee of the Faculty of Health Sciences at the University of Cape Town (REC Ref:247/2013). Ethical approval for the PRIME research programme has also been obtained by local ethics committees in study countries. All those who participated in the semi-structured interviews gave informed consent to participate.

### Findings

Between two and four ToC workshops were held in each country at different levels of the health system to develop ToCs for the PRIME district site, with a total of 13 workshops across the 5 countries. Table 2 outlines how each workshop was structured and Table 3 describes the stakeholders who participated in the workshops. More detail about the structure, stakeholders and how the workshops were conducted to achieve their multiple goals are described in subsequent sections.

**Table 2:** Summary of PRIME ToC workshops

**Country**	**Level**	**Location**	**Length**	**Structure**
**Ethiopia**				
**Workshop 1. (ET1)**	Community and district level representatives	Sodo*	½ day	a Introduction to PRIME
				b Explanation of the ToC process
				c Agreement on impact
				d Worked forwards to development of the ToC discussing current services, needs and potential outcomes of the ToC to reach the desired impact.
**Workshop 2. (ET2)**	National level planners	Addis Ababa	1 day	a Introduction of Ethiopian mental health strategy by national ministry of health representative
				c Introduction to ToC and the ToC process
				c Review and refinement of the ToC developed in ET1.
**India**				
**Workshop 1. (IN1)**	District and health Facility	Sehore*	1 day	a Introduction to mental health and PRIME
				b Introduction to ToC
				c Mental health presentation
				d Group work where each group developed the outcomes pathway for the ToC
				e Feedback from group work
**Workshop 2. (IN2)**	District and health Facility	Sehore*	1 day	a Summary of IN1
				b Group work: details of interventions and assumptions at community, health facility and health organisation level in the existing ToC map.
				c Presentation and discussion of the integrated mental health care plan developed from the ToC.
**Nepal**				
**Workshop 1. (NE1)**	Health Facility and District	Chitwan*	1 day	a Introduction to PRIME
				b Introduction to ToC
				c Agreement on long-term impact and worked the group agreed on the long term impact then worked backwards to determine the outcomes, interventions and assumptions needed to achieve this.
**Workshop 2. (NE2)**	National level planners	Kathmandu	½ day	a Introduction to PRIME
				b Introduction to ToC
				c ToC from NE1 was presented, reviewed and refined by the group.
**Workshop 3. (NE3)**	Health Facility and District	Chitwan*	½ day	a Review of the ToC developed in NE1 and NE2
				b Discussion of potential adaptation for specific disorder and indicators to measure outcomes.
**Workshop 4. (NE4)**	National level planners	Kathmandu	½ day	c Review of the ToC refined in NE3
				d Discussion of potential adaptation for specific disorders and indicators to measure outcomes.
**South Africa**				
**Workshop 1. (SA1)**	Health facility, district, provincial and national level representatives	Dr Kenneth Kuanda*	2 days	a Introduction to PRIME
				b Introduction to ToC
				c Used part of the PRIME cross country ToC and worked forward adding detail to each outcome for all four disorders.
**Workshop 2. (SA2)**	Community	Dr Kenneth Kuanda*	1 day	a Introduction to PRIME
				b Introduction to ToC
				c Used part of the PRIME cross country ToC and worked forward adding detail to each outcome for all four disorders.
**Workshop 3. (SA3)**	Health facility, district, provincial and national level representatives	Dr Kenneth Kuanda*	1 day	a SA1 workshop was reviewed briefly.
				b Disorder specific integrated mental health care plan based on SA1 was presented and discussed in detail.
				c PRIME evaluation plan and next steps were discussed.
**Uganda**				
**Workshop 1. (UG1)**	District and health facility level	Kamuli*	1 day	a PRIME, mhGAP, challenges for mental health care and the ToC were introduced.
				b The impact was agreed on and the group worked backwards to develop the ToC.
**Workshop 2. (UG2)**	District and health facility level	Kamuli*	1 day	a The group was oriented to the ToC process, PRIME and planned work.
				b The ToC map from UG1 was reviewed and refined.

Locations marked with * indicate the PRIME district in the respective countries.

**Table 3:** Number and category of workshop participants in the ToC workshops

**Country**			**Ethiopia**		**India**		**Nepal**				**South Africa**			**Uganda**	
**Abbreviation**			**ET1**	**ET2**	**IN1**	**IN2**	**NE1**	**NE2**	**NE3**	**NE4**	**SA1**	**SA2**	**SA3**	**UG1**	**UG2**
**Number of participants**			17	13	20	17	14	10	11	8	38	26	37	22	22
**Category of stakeholders**															
**1.**	**Policy makers**														
	National* Health representatives			*X				X		X	X*		X*	X*	X*
	State/province Health representatives				*X	*X					X		X		
**2.**	**District level planners and management**														
	District Health representatives														
		Health planners/managers	X	X	X	X								X	X
		District Medical officers			X						X		X		
		MH coordinators				X	X		X		X	X	X		
		Other health coordinators					X		X		X			X	X
		Other district administrative or finance staff	X											X	X
		Other district representatives (Justice, Education)	X												
**3.**	**Specialists**														
		Psychiatrists	X*	X	X	X	X		X	X	X		X		
		Psychologists			X	X	X	X	X	X	X		X		
		Psychiatric clinical officers												X	X
		Psychiatric nurses						X						X	X
	Other Medical Specialists										X		X		
**4.**	**Service providers**														
	Community health center, primary health center and sub health posts														
		Clinic managers									X		X		
		Medical officers			X	X	X		X		X		X		X
		Clinical officers												X	
		Health Assistants					X		X						
		Nurses					X		X					X	
		Lay Health workers (clinic based)					X		X		X	X	X		X
		Lay Health workers (community based)											X		
		Other clinic staff			X	X								X	
**5.**	**Researchers**														
		PRIME	X	X	X	X	X	X	X	X	X	X	X	X	X
**6.**	**Community and civil society**														
	NGO/development organisations		X		X	X		X		X	X	X	X		
	Community														
		Community leaders	X										X		
		Media		X											
		Faith leaders	X									X			
		Traditional healers										X			
**7.**	**Mental Health Service users**							X		X		X			

*Members of the PRIME Consortium.

### ToC Workshop structure

The ToC workshops were structured to include a welcome, a brief introduction to PRIME, a discussion on mental health (in some cases this was discussed in a pre-workshop meeting) and an introduction to the ToC approach. In India, Uganda and Ethiopia, the workshops were introduced by state or national Ministry of Health representatives.

Most countries started from the impact and worked backwards to map long, medium and short term outcomes required to achieve the impact. When developing the initial TOC map, workshop participants in all countries except South Africa developed a generic ToC map for mental illness rather than developing separate ToCs for each priority disorder (depression, alcohol use, psychosis and epilepsy). This was because the workshop facilitators hypothesised that the causal pathway through which the integration of mental health into primary care leads to improved outcomes were essentially the same for different disorders. The generic map was then compared and modified for the different disorders with very few changes needed for the specific disorders.

### Stakeholders

The stakeholders were selected using a variety of criteria including: 1) involvement in planning or implementing the PRIME MHCPs at various levels including providing specialist care, co-ordinating services, providing primary care services, managing facilities or developing and evaluating the MHCPs; 2) membership of PRIME Community Advisory Boards who provide an oversight function to PRIME at district level; 3) specialist knowledge of mental health; 4) representatives of service users or other sectors in the wider community; or 5) decision making power or control over resources.

The size of the workshops varied considerably across countries with a median of 15 (Interquartile range 13 – 22) stakeholders attending each workshop (Table 3). Most countries held preliminary and final workshops with the same group of people, comprising stakeholders at different levels of the health system (Table 2) with the exception of Ethiopia where their first workshop included district representatives and the second national level representatives and mental health specialists. Some countries, such as Uganda, India and South Africa relied on key individuals to assist with the identification of participants. These were often the District Medical Officers who assisted by inviting the participants to the workshop and thus providing the workshop with local legitimacy.

The stakeholders attending the workshops varied by country and by workshop (see Table 3). However, five key groups of stakeholders attended all workshops: policy makers; district level health planners and management; mental health specialists; researchers; and service providers. These groups were not mutually exclusive and many stakeholders belonged to more than one category. Some countries also had representation from community or non-governmental organisations (NGOs) but there was very little mental health service user representation with only 3/13 workshops including mental health service users.

A major potential barrier to stakeholder participation in the workshops was the hierarchical nature of local health service organisation which would have inhibited participation by lower level staff. Consequently, the PRIME country teams who facilitated the workshops attempted to mitigate the effect of the hierarchical structures by stratifying the workshops and holding separate workshops at different levels of the health system or by limiting the levels of the participants in the workshops. For example, the Nepalese and Ethiopian facilitators stratified their workshops into district and national level workshops while South Africa held a separate workshop at the community level. Indian facilitators specifically chose to limit their workshop to district level and senior facility level in order to prevent power differentials and to optimise planning. According to one of the facilitators, this resulted in everyone “*participating because there was no hierarchy, they were all district level, all sub district level officers*". This is in direct contrast to the start of the workshops where senior district and state level stakeholders were asked to open the workshop: *“when the four of them were in the room, I think no-one was speaking anything, they cannot, I mean even if they wish to they cannot speak… once all these four people were out and then all of a sudden everyone was speaking*”.

### Achieving the goals of the TOC workshops

The ToC workshops had 3 main goals: development of a ToC map to reflect the structure of the proposed district MHCP; contextualisation of the MHCP; and ensuring the engagement and buy-in of key stakeholders to the MHCP. We describe how these goals were achieved below.

#### 1) The development of a structured, logical and evidence-based ToC map

The ToC workshops helped to develop a structured and logical ToC which was described by facilitators as a “*visual map*” which, “*like a map of the city*”*,* they could refer to when thinking about their MHCP.

In four of the five countries, the ToCs were developed during the workshop with stakeholders agreeing on the intended impact of the PRIME MHCPs and then working backwards to determine the outcomes needed to achieve this impact. In South Africa, instead of developing a ToC from scratch, facilitators used the basic building blocks of the cross country ToC to initiate the discussion. They asked stakeholders to comment on the validity of this ToC and then used this to elicit more detail from the stakeholders.

Facilitators reported the process of working with the group to map out the long, medium and short term outcomes which helped to reach consensus as they had to work with stakeholders to “*refine and redefine and,… eventually agree*”. It also encouraged facilitators and stakeholders to focus on outcomes rather than interventions. This was a change from usual practice, as one facilitator from India observed: “*most of us in the field of development… or public health [are] very focused on … interventions and activities”*. Assumptions underlying the ToC maps were discussed in all countries, however the primary focus was, as a Ugandan facilitator noted, more *“on the process and the outcome more than… the assumptions*”.

The rationale, or evidence base, underlying the pathways on the ToC map, and the indicators used to determine success, were seen as the domain of the researchers. When the rationale was discussed, it was only in relation to whether interventions would be required. Most facilitators thought that sourcing the evidence base underlying the ToC was the role of the researchers, as a Ugandan facilitator noted, “*because, that’s about literature, evidence, and that is for us really”.* Similarly, some countries discussed indicators in their final workshop, however, this was seen as something which was more important for the researchers: *“it’s very important for [the researchers] to know that everything has been covered and to be able to evaluate the plan using indicators”* although it may not be *“necessary for everyone who was attending that ToC workshop”.* Therefore many country teams added the indicators once the workshops were completed.

The planned interventions were discussed in all country workshops. Some countries focused on this in detail and added additional elements to the ToC workshops. In India, South Africa and Ethiopia, facilitators probed more into the resources required to implement the MHCP and the roles and responsibilities of service providers in the intervention. As one South African facilitator explained,

“*using the TOC process is really important …… in order to be able to enact the TOC plan, we need these resources in place and this is what each of these resources are going to be doing and this is how we’re going to capacitate them in order to fulfil their roles and responsibilities*”.

This was particularly important in many PRIME countries as no new human resources were being made available to implement the plan, apart from those already available in the district.

In South Africa and India, facilitators provided additional detail during the final workshops by presenting an integrated mental health care plan based on the preliminary ToC workshops. A facilitator from India noted that *“participants were very happy to see that what they had done, in the first workshop… came out in a very refined manner and very systematic manner".* A South African facilitator reported a similar experience,

“*we came on board with stuff that people had already discussed and agreed on and there was some clarifications that were made, a few additions that were made and in essence when we came to the workshop, there was already agreement that had been reached with the previous workshop so this is just consolidating what we had on paper and I felt that everybody was moving in the same direction*".

#### 2) Contextualising the mental health care plans

The second purpose of the workshops was to ensure that the ToC and the MHCP were contextually relevant. During the workshops, the researchers gained contextual knowledge in several domains including the functioning of district health services, planning for mental health programmes, physical resources, medication provision, human resources, stigma, cultural understanding of mental illness and the existing community structures.

Stakeholders identified challenges, needs and potential solutions. For example, the provision of psychotropic medication was identified as a challenge in the Nepal district level workshop. Although a steady supply of psychotropic medication is necessary for effective treatment of severe depression and psychosis, no antipsychotic medications are on the free drug list and supply of medications is irregular with frequent stock outs. Policy makers at the national level workshop were able to provide potential solutions to this problem including agreeing to provide psychotropic medications in the area of implementation of the PRIME MHCP and suggesting that additional stock is ordered as a buffer and procurement processes for emergency supplies are put in place. Similarly, during the final workshop in South Africa, stakeholders identified the need for psychologically trained supervisors for lay counsellors providing psychosocial interventions. They suggested that intern psychologists could be made available in the short term with a long term view to lobby the Department of Health to create new posts for graduates with a Bachelor of Psychology in Counselling degree (BPsych Counselling).

#### 3) Obtaining stakeholder buy-in

The third purpose of the workshops was to obtain buy-in from the stakeholders on the ToC and MHCPs. As one facilitator remarked, one can *“have a beautiful TOC map but if [one doesn’t] have the buy-in and … the various human resources available to make it work it’s not… going to work".* Buy-in was achieved through the process of developing the map in consultation with stakeholders. This resulted in a sense of ownership where “*the people who were there in both the workshops feel that it’s their product*”. This buy-in, where “*the district has owned the theory of change”,* was felt to be an important contribution of the workshops. A South African facilitator noted, *“the most important thing to derive from that second workshop was to get consensus and agreement from particularly the decision makers about who would do what… we need people to buy into…these new roles and responsibilities because it does mean quite a shift.”* However, facilitators noted that this buy-in may not be achieved if used to cover a larger population. One facilitator from India cautioned that *“a lot of effort need to be taken before theory of change can be used as a routine tool for scaling up of programme planning”.*

There was general recognition that buy-in was necessary from those who were in “*positions of decision making and affect availability of services and resources”.* But, as a Ugandan facilitator noted, this buy-in may be that of individuals who “*may not have the power or the political will to change what you need changed*.” One facilitator cautioned that it was important to have “*both a top down and bottom up approach*” where “*political credibility*” was provided by national and state representatives and service providers “*being part of that process was really important”* so that they could *“see that it will actually be part of their work that they do*.” However, it was often not possible to have all stakeholders present.

### The contributions of stakeholders to the goals of workshops

The five key groups of stakeholders who contributed in each country to at least one of the workshops (mental health specialists, researchers, policy makers, district level health planners and management, and service providers), contributed in different ways to achieve the three goals of the ToC workshops.

Mental health specialists and researchers provided details on technical issues such as functioning of existing mental health care provision in the district, the need for prioritisation of additional disorders (such as epilepsy in Nepal) and the provision of feasible and evidence based interventions. The researchers, who were facilitating the workshops and were often also mental health specialists, provided the technical knowledge of the ToC process to develop a logical evidence based map. They also provided much of the evidence base underlying the interventions and the indicators for the ToC which were often developed after the workshop.

The policy makers and national level planners made higher level contributions on the structure of the MHCP and possible solutions to issues such as medication procurement in Nepal and supervision structures in Ethiopia. They did not provide much additional information on the structure of the ToC map or the details of the MHCPs when separate policy maker workshops were held in Nepal and Ethiopia as these had been provided in the district level workshops: *“there wasn’t much when it comes to high level …there weren’t many changes from the first one, it was like… reaching a point of saturation*." Buy-in from policy makers both prior to and during the workshops was essential as they control resources, for example “*they are responsible for planning all … health care”* and *“allocate budget and programme in government health system*.” The support of policy makers, who often introduced the workshops when countries only held them at one level, was seen as a way of legitimizing the PRIME project and the ToC workshop. They provided “*political credibility*” and, in some cases such as India and Uganda, were the reasons the facilitators felt that the workshops were so successful.

District level health planners and management were the main contributors to developing the overall structure of the ToC map in most of the workshops as well as providing contextual information on what they felt could or could not work. This included identifying current challenges, needs and potential solutions. For example, in Nepal they identified constraints such as incentive structures for volunteers and medication shortages, whereas stakeholders in Uganda identified the low priority of mental health and the stigma towards service providers who work with people with mental illness. In the South African and Ethiopian workshops they identified additional community workers who could potentially be utilised for PRIME.

Service providers assisted with providing detailed information about the context and the functioning of existing systems including current workloads of personnel. As such they could comment on their ability to take on additional tasks envisaged by the MHCP. As described above, their input and buy-in was seen as essential as they would predominantly be providing the services outlined in the MHCP.

The contribution of stakeholders to the workshop was moderated by the presence of other stakeholders who were considered higher up in the health system hierarchy. This depended on the strength of the hierarchy which was considered particularly strong in Nepal and India. This led a facilitator from India to remark that the *“idea that ToC could involve everyone from health policy makers to planners to providers to community health workers in one session…needs to be kind of retested because it cannot be participative in government structures which run on hierarchy”*.

## Discussion

In this study, we describe how district specific ToC workshops were used to plan for the integration of mental health services into primary health care in five low resource settings. Comparing workshops across the five countries working in PRIME has allowed us to distil some key lessons on the use of ToC workshops for complex mental health intervention development and reflect on these in relation to existing literature.

We found that ToC workshops provided a useful approach to developing a logical structure for mental health plans and provided contextual details for implementing these in district sites and obtaining stakeholder buy-in. The participatory nature of the ToC workshops allowed stakeholders to work together in a structured forum to map out the ToC for the district and creating a forum for knowledge exchange and dialogue about needs and potential solutions.

In this process, the power relationships between the stakeholders was critical, as confirmed by previous research that shows that all actors within health services can exert different power over implementation of health policy [35] and that service providers may choose to exercise this power to both promote or hinder implementation [36]. Therefore the active participation and buy-in of all stakeholders is likely to increase the chances of successful implementation [6]. This is particularly important in the context of mental health services in LMIC where stigma is high [37], human resources for health are limited [38], funding is minimal [2] and political priority is low [1].

From the outset it was clear in some countries that hierarchies within the health system would make it difficult for district level planners and service providers to participate despite using facilitation techniques. As their input was seen as essential to the process of the development of the ToC and contextualising the MHCP, country teams used various strategies to mitigate these hierarchies. These included: 1) stratification of stakeholders by having separate workshops for service providers and for policy makers; 2) limiting participants to a homogenous group of stakeholders; or 3) seeking high level buy-in in other forums, for example, interviews. Although these strategies seem to have increased participation, the ToC is no longer ‘owned’ by all potential stakeholders as is recommended by the Aspen Institute [39]. This is similar to the finding by Sullivan H and Stewart M [40] that it may not always be feasible to achieve total ownership of a ToC where all stakeholders are involved in the planning and development of a ToC. They propose that ToCs may be owned by different groups of stakeholders including the evaluators, by a dominant stakeholder, the community or an elite group of implementers.

Other aspects of the ToC process also reduced the ownership by all potential stakeholders such as the lack of beneficiaries of the program as well as the finalisation of the ToC by the PRIME researchers. Mental health service users were present in only 3 of the 13 workshops. Although most facilitators would have liked to included mental health service users as beneficiaries of the programme who can provide an alternative perspective on mental illness and care [41,42] they were not included in most workshops because there are currently limited or non-existent mental health services in PRIME district sites and no active advocacy groups for mental health service users [30]. PRIME researchers were involved in finalising some aspects of the ToC after the workshop such as the rationale and indicators without involving the whole group of stakeholders included in the ToC. Therefore, despite including quite a broad range of stakeholders (see Table 3), the ownership of the ToCs in PRIME countries most closely resembles what Sullivan and Stewart (2006) refer to as elite ownership of the ToC: ownership by a small group of leaders including community leaders who are involved in setting up and implementing the program. Sullivan and Stewart (2006) propose that the ToC process might still be effective as these stakeholders often have access to significant resources needed to support and implement wider systematic change.

It is difficult to ascertain post-hoc whether it would have been possible in these settings to run a workshop with all identified stakeholders or how the stakeholder composition of the workshops has affected the resultant quality and validity of the ToC. Certainly, the inclusion of multiple levels of stakeholders in the ToC workshops enabled a combination of top down and bottom up approaches to planning by either acting as a structured forum for discussion where all stakeholders participated in the same workshop or as a conduit between policy makers and service providers where workshops were stratified. The ToC workshops enabled district level stakeholders to directly influence the planning process which was then vetted by the policy makers who agreed to implement the plan. Undoubtedly the initial success PRIME has had in facilitating the bottom up planning process was directly influenced by the participation of Ministry of Health partners in the consortium formalised through Memoranda of Understanding and on-going policy engagement [27]. However, it is yet to be established whether this participatory process has resulted in real ownership of the MHCPs on the ground by service providers and a real increase in resources from senior policy makers.

A key limitation of the workshops was the lack of explicit focus on the assumptions underlying the ToC in the workshops. Assumptions are seen as one of the core elements of ToC which allow stakeholders to ensure that they understand each other’s perspectives [12]. These were not covered in detail, as the facilitators wanted to focus more on the outcomes and interventions and felt the assumptions may have been too complex for some of the stakeholders. However, the rich discussions reflected in the content of the workshops indicated that assumptions did emerge during the discussions between stakeholders.

There were several shortcomings of this study. First, our sample size was small and we focused only on the experiences of workshop facilitators, both in the interviews and in the process documentation produced by research teams. Some of these facilitators were also included as authors on this paper and the remaining authors were involved in supporting the facilitators in conducting and refining their ToCs. This may result in a biased view of the ToC workshops and an overestimation of their usefulness. In future it would be useful to gauge the extent of buy-in from other stakeholders and examine this with the ToC process over the course of the project to determine the impact of the long term influence of the ToC process. Secondly, we did not explicitly examine the power relationships within the workshops. For example, the researchers may have been seen as powerful “experts” within the field which may have prevented frank discussion amongst stakeholders and a social desirability bias in the workshop participants. Thirdly, we did not explore the roles and contributions of mental health service users to the ToC process which are likely to have been different from other stakeholders. Finally, this paper focused on a small aspect of the ToC process within PRIME, namely the district specific ToC workshops. A more detailed description and analysis of the overall ToC process within PRIME, including the role of the ToC in the development of the PRIME MHCPs and the evaluation design is necessary and planned in a subsequent paper. Despite these limitations, we were able to draw a rich comparison of experiences across countries who had quite similar experiences across settings and draw on some key lessons for conducting ToC workshops within the health system in LMIC:

1. The goals of the workshops should be clearly stated prior to the workshop. This should include a statement about the level of detail required in the workshops and resulting ToC, as well the ideal ownership of the ToC and potential limitations thereof.

2. The number, length, structure, components of the ToC and stakeholder composition should be flexible and adapted to ensure the ToC workshops can meet the stated goals within the context.

3. Facilitators need to be aware of the health system hierarchies and composition of workshops should be balanced to manage these using facilitation or stratification to ensure the ToC can meet the stated goals.

4. Additional strategies such as individual interviews or reviews of the resulting ToCs may be necessary to involve stakeholders not included in the workshops to ensure broader ownership of the ToC.

5. The support of policy makers is important throughout the process to add legitimacy to the workshops and increase the likelihood of implementation of the resulting MHCP.

## Conclusions

This study has shown how ToC workshops can be conducted to develop ToCs as a basis for contextualised district level MHCPs and to facilitate stakeholder buy-in. The ToC workshops in PRIME demonstrated that different stakeholders contribute different perspectives to the planning process and although a wide range of stakeholders should be included, hierarchical health systems may limit the participation of all stakeholders in the workshops. Various strategies may be required to mitigate these effects to achieve the stated goals of the workshops. However, these may limit the ownership of the ToC.

## Competing interests

The author(s) declare that they have no competing interests.

## Authors’ contributions

The study was conceptualised by EB under the guidance of CL and MDS. AF, IP, JN, VM and NL conducted the ToC workshops in PRIME countries and contributed to the collection of process documentation. EB conducted the interviews and data analysis and drafted the manuscript under the guidance of MDS and CL. All other authors were involved in critically revising the manuscript and all authors approved the final draft before publication.
